# Editorial: Association of diabetes mellitus with cognitive impairment and neurological disorders

**DOI:** 10.3389/fendo.2024.1505230

**Published:** 2024-10-18

**Authors:** Joana Nicolau, Antelm Pujol

**Affiliations:** Vascular and Metabolic Diseases Research Group, Endocrinology Department, Son Llàtzer University Hospital, Health Research Institute of the Balearic Islands (IdISBa), Palma de Mallorca, Spain

**Keywords:** diabetes mellitus, cognitive impairment, type 1 diabetes, electroacupuncture, phytate, mild cognitive impairment, retina

The intersection of diabetes mellitus (DM) and cognitive impairment (CI) has become a critical area of research due to the rising prevalence of diabetes globally and their associated neurocognitive complications. This editorial aims to frame the research findings within this Research Topic by exploring the relationships between diabetes and cognitive decline, neurovascular asymmetry, underlying physiological mechanisms and future treatments options highlighted in the contributing articles. Each study adds valuable insights into how diabetes, both Type 1 and Type 2, affects cognitive function, posing significant public health concerns.

Tassew et al. systematically reviewed the prevalence of CI among patients with DM in Africa, finding a pooled prevalence of 43.99%. Key factors associated with CI included primary education level, poorly controlled diabetes, age over 60, and diabetes duration of more than 10 years. This review aimed to assess these associations and provide insights into the burden of cognitive dysfunction in populations with DM. Their findings highlight the need for an early diagnosis and targeted healthcare interventions to address CI in patients with DM, particularly those at higher risk due to these contributing factors mentioned before.

Moreover, having effective and low-cost screening tools is essential for early diagnosis. Lei et al. developed and validated a predictive model for cognitive dysfunction in individuals with abdominal obesity by using factors such as age, sex, education, total fat intake, red blood cell folate, depression, and physical activity. The model was built using data from 1,490 participants and assessed for predictive accuracy. Results showed strong predictive performance, with C-index values of 0.814 for the training set and 0.805 for the validation set. The nomogram effectively identified individuals at risk for cognitive dysfunction, demonstrating good clinical utility for early intervention and risk management.

In this regard, there may be additional diagnostic tools beyond cognitive tests. Some studies suggest that the retina may serve as a window to the brain. In fact, Li et al. explored the relationship between mild cognitive impairment (MCI) and retinal nerve fiber layer (RNFL) thickness in type 2 diabetes mellitus (T2DM) patients. RNFL was assessed by using optical coherence tomography (OCT). Serum levels of IL-18, irisin, CML, and RAGE were also determined. Their results showed that T2DM patients with MCI had thinner RNFL and higher levels of IL-18, CML, and RAGE, whereas irisin levels were reduced. These markers were significantly correlated with cognitive test scores, suggesting their potential use as diagnostic indicators for MCI in T2DM patients.

Furthermore, not just retinal nerve fiber layer, but other brain changes have also been described. Samoilova et al. evaluated interhemispheric asymmetry in brain structure, metabolism, and neurovascular changes in patients with type 1 diabetes and cognitive impairment. By using MRI, spectroscopy, and perfusion imaging, their research revealed a significant asymmetry, particularly in frontal and occipital lobes. White and gray matter atrophy, along with metabolic disturbances in the hippocampus, were identified. These changes correlated with cognitive decline, particularly in attention and memory. Their findings suggest that neurovascular and metabolic alterations contribute to cognitive impairment in type 1 diabetes, highlighting the importance of early detection through neuroimaging.

Besides, an early diagnosis could present a unique opportunity to reverse brain structural and connectivity changes. In fact, Fang et al. explored cognitive reversal and brain connectivity changes in young adults with T2DM. Participants showed cognitive improvement in areas like global cognition and executive function after 18 months of a proper glycemic control. Brain connectivity, which was enhanced at baseline, normalized over time, and this reduction was linked to cognitive gains. This study suggests a potential “window period” for reversing cognitive dysfunction in early-stage diabetes. However, no clear association between glycemic control and brain connectivity was found, highlighting the complexity of the relationship between blood glucose and brain function.

Also, some emerging therapies have been proposed. Li et al. evaluated the effects of electroacupuncture (EA) on cognitive function and metabolic disorders in Alzheimer’s disease (AD) model mice. EA improved cognitive abilities, reduced tau phosphorylation, and enhanced neuronal morphology. Additionally, EA regulated metabolic disorders by promoting brown adipose tissue (BAT) thermogenesis and improving peripheral glucose and lipid metabolism. EA also reduced insulin resistance and increased insulin sensitivity in the brain. These findings suggest that EA has therapeutic potential for treating AD by addressing both cognitive deficits and underlying metabolic imbalances, particularly through BAT activation and central insulin pathway regulation. In the context of human clinical trials, Pujol et al. proposed a promising clinical trial protocol which aimed to assess the effects of daily phytate supplementation on the progression of MCI, brain iron deposition, and diabetic retinopathy in T2DM patients. Over a 56-week randomized, double-blind, placebo-controlled trial, cognitive changes, brain iron accumulation using MRI, and retinal health will be evaluated. Their hypothesis is that phytate supplementation could improve cognitive function, reduce iron accumulation in the brain, and slow neurodegeneration in both the central nervous system and the retina, potentially offering a new therapeutic strategy for T2DM patients with MCI.

In conclusion, the association between diabetes mellitus and cognitive impairment represents a growing public health challenge due to the global increase in both conditions. All these studies offer valuable insights into the underlying mechanisms of this relationship, from the importance of early detection to emerging therapeutic innovations. There is an urgent need for personalized medical interventions, accessible diagnostic tools, and a comprehensive approach that addresses both metabolic disturbances and neurological symptoms to mitigate the impact of cognitive decline in patients with DM.

